# Predictors of Coping Strategies in Caregivers of Family Members with a Brain Tumor: A Correlational Study

**DOI:** 10.3390/healthcare12181897

**Published:** 2024-09-21

**Authors:** Hsiang-Hua Lu, Shu-Yuan Liang

**Affiliations:** 1 School of Nursing, National Taipei University of Nursing and Health Sciences, 365 Ming Te Road, Beitou, Taipei 112, Taiwan; 2 Department of Nursing, National Taiwan University Hospital, Zhongshan South. Road., Zhongzheng District, Taipei 100, Taiwan

**Keywords:** brain tumor, caregiver, caregiving competence, coping strategy, family function

## Abstract

Background/Objectives: Brain tumor patients confront numerous challenges arising from diagnosis and treatment, and these impact the patient’s physical, mental, and social functions at all levels. Primary informal caregivers assume a pivotal role in home-based patient care. Of particular importance are the coping strategies employed by family caregivers, as they can influence both their own health and the overall quality of home care. This study aimed to explore the associations among family function, caregiving competence, and coping strategies among primary informal caregivers. Methods: This study adopted a cross-sectional correlational design and convenience sampling to survey the primary informal caregivers of 111 brain tumor patients. The study instruments included the Family Assessment Device General Function, Caregiving Competence Scale, and Revised Ways of Coping Checklist. Results: The findings of this study revealed a significant positive correlation between the family function of primary informal caregivers and their employment of emotion-focused coping (r = 0.209, *p* < 0.05). Furthermore, caregiving competence exhibited a positive association with problem-focused coping (r = 0.242, *p* < 0.05) and emerged as a significant predictor of problem-focused coping (β = 0.182, *p* < 0.05). However, neither family function (r = 0.059, *p* < 0.05) nor caregiving competence (r = 0.031, *p* < 0.05) demonstrated significant associations with total coping strategies. Conclusions: The findings of this study affirmed that enhancing the caregiving competence of primary informal caregivers of brain tumor patients can facilitate the adoption of problem-focused coping strategies.

## 1. Introduction

In the United States, cancer is the second leading cause of death [1]. There are approximately 23,820 new cases of brain or central nervous system tumors reported each year, resulting in 17,760 annual fatalities [2]. Meanwhile, in Taiwan, cancer ranks as the leading cause of death. In 2021, there were 756 cases of primary malignant brain tumors, comprising 0.62% of all malignant tumor cases. Nevertheless, brain tumors accounted for 1.14% of all malignant tumor-related deaths [3].

Although the incidence rate of brain tumors is not the highest among the cancers, the compression of brain tissue by tumors or related treatment can cause neurological damage, which may cause aphasia, visual impairment, and cognitive function impairment and may further affect the patient’s physical activity [4,5,6,7]. The main treatment methods for brain tumors include surgery, chemotherapy, and radiation therapy. The related side effects of treatments not only have an impact on the patient but also affect their primary informal caregiver’s coping strategies on issues related to home care [8]. Primary informal caregivers play an important supporting role in the care of patients at home. Therefore, it is important to understand how primary informal caregivers cope with the stressful situations arising from caring for brain tumor patients at home.

Coping strategies are defined as an individual’s “constant cognitive and behavioral efforts to manage specific external and/or internal demands that are perceived as taxing or surpassing the person’s resources” [9]. Coping strategies reflect the choices made by family members following an evaluation of the stress, such as employing problem-focused coping or emotion-focused coping. The outcomes of these coping strategies can significantly impact the perceived stress levels and psychological well-being of family members [10,11]. Additionally, they can influence the quality of care provided to family members with cancer [12]. Family caregivers may encounter various challenges in home care situations [13]. When tending to an ill family member, the caregiving competence and family function of caregivers may play pivotal roles in shaping their coping strategies [10,14,15].

Caregiving competence refers to the positive response to caregiving responsibilities, reflecting an individual’s growth in their caregiving role [16]. This competence can significantly influence the coping strategies adopted by primary informal caregivers [10,14]. Previous research has revealed that, in addition to providing physical care, family members’ caregiving tasks for cancer patients also encompass offering emotional support, managing unexpected patient situations, seeking relevant resources, and making decisions regarding caregiving activities. Throughout this process, family members may encounter various challenges and forms of resistance [13], emphasizing the importance of possessing caregiving competencies to effectively perform tasks essential for implementing coping strategies. Previous studies demonstrated a significant association between the caregiving competence of primary informal caregivers and their coping strategies [10,14]. Notably, caregiving competence plays a pivotal role in fostering problem-focused strategies among family members [14]. The family serves as the core and enduring source of social support for individuals. However, the demands of caregiving tasks can disrupt the balance of family members’ original social roles and the dynamics of family functioning, potentially influencing their coping strategies when faced with various caregiving pressures [15]. 

Family function refers to the emotional bonds among family members, established family rules, communication patterns within the family, and the effectiveness of the family system in responding to external events [17]. It is associated with the strengths and weaknesses of the family structure, as well as indicators of interaction among family members [18]. Family function influences individuals’ coping with stressful events [19,20,21,22]. Therefore, the health status of the family function (healthy or unhealthy) may affect the coping strategies of primary informal caregivers when performing caregiving duties. Previous research revealed that unhealthy family functions, such as low cohesion, low support, high control, and highly restrictive family structures, may lead individuals to adopt avoidant coping strategies. Conversely, healthy family functions, including high cohesion, high support, and low conflict family structures, tend to encourage individuals to adopt constructive coping or active coping [20,23,24,25]. Moreover, in families with open communication and clear role assignments, individuals are inclined to utilize problem-focused coping to address their challenges [26]. Furthermore, family function can significantly predict individual coping strategies [15], and families play a crucial role in providing social support. However, in the field of cancer care, there is limited research exploring the impact of family function on the coping strategies of primary informal caregivers.

Families of brain tumor patients experience diverse caregiving pressures at home. The coping strategies employed by family caregivers significantly impact the quality of care provided to relatives with cancer. Consequently, comprehending these coping strategies and their predictors in caring for family members emerges as a crucial topic. Such understanding can further assist professional caregivers in identifying avenues to enhance coping strategies among family caregivers.

This study examined how primary informal caregivers’ coping strategies relate to their caregiving competence and family function specifically for brain tumor patients, a focus often overlooked in broader cancer research. Although the impact of caregiving on psychological well-being is well documented, research is lacking on how family dynamics and caregiving competence affect coping strategies for brain tumor informal caregivers. This study addressed this gap by investigating the influence of caregiving competence and family function on informal caregivers’ coping strategies, aiming to enhance support for caregivers and improve patient care. Hence, the aim of this study was to explore the relationships among family function, caregiving competence, and coping strategies among primary informal caregivers and to identify predictors of their coping strategies.

## 2. Materials and Methods

### 2.1. Study Design

This study utilized a cross-sectional correlational research design and convenience sampling to explore the associations among family function, caregiving competence, and coping strategies among primary informal caregivers of brain tumor patients. The Strengthening the Reporting of Observational Studies in Epidemiology (STROBE) guidelines were used to aid in the presentation of the observational study, ensuring that the study was conducted and reported adequately [27].

### 2.2. Samples, Setting, and Procedure

A total of 111 primary informal caregivers of hospitalized brain tumor patients were recruited for this study. This study utilized G*Power software [version 3.1, Heinrich Heine University, Dusseldorf, Germany] to estimate the sample size. The main statistical method employed was multiple linear regression analysis. The effect size was set at 0.20, the α value was set at 0.05, and the desired power was set at 0.80. Additionally, the parameter settings included a total of sixteen variables. The estimated sample size required was 111 participants. Structured questionnaires were administered to collect data. Participants were recruited from the neurosurgery ward of a medical center in northern Taiwan during the period from April 2021 to August 2021. The inclusion criteria for this study were as follows: (1) being a family member of patients diagnosed with primary malignant brain tumors or metastatic brain tumors; (2) aged 20 years or older and related to the patient by blood or marriage; (3) designated as the primary informal caregiver by the patient; (4) ability to communicate in Chinese or Taiwanese; and (5) providing care for brain tumor patients for at least one month.

The research subjects of this study were inpatients and their families. The principal investigator (author Lu) selected eligible subjects from medical records, explained the purpose of the study and how it would be conducted, and then sought the consent of the patients and their families to participate. After obtaining consent, a consent form was completed, and the family members chose a convenient time for the principal investigator (author Lu) to conduct a structured questionnaire survey. This structured questionnaire included basic demographic information about the family members, a family function scale, a caregiving competence scale, and a coping scale. After the participants completed the structured questionnaire, the investigator reviewed the documents to identify any omitted information. Participants were then invited to provide any missing details. Additionally, the investigator collected information on medical characteristics of patients from the patients’ medical records.

### 2.3. Ethical Considerations

This study obtained approval from the Institutional Review Board of a medical center of National Taiwan University Hospital in northern Taiwan (approval trial number: 202012062RINC, approval trial date: 2 March 2021). The survey was conducted by the principal investigator (author Lu), who verbally explained the study’s purpose and procedures to potential participants, namely, primary informal caregivers, to seek their consent. Participants were asked to sign a consent form indicating their willingness to participate. To protect participants’ privacy, the questionnaire employed codes instead of requesting personal data. During data collection, if primary family caregivers declined to complete the questionnaire or expressed unwillingness to continue, the investigator would respectfully conclude the questionnaire based on their preferences.

### 2.4. Measurements

#### 2.4.1. Sociodemographic Variables

The data collection involved gathering information on various aspects of primary informal caregivers, including their age, gender, religious affiliation, education level, marital status, health status, and employment status. Additionally, data were collected on their household annual income, relationship with the patient, type of caregiving provided, duration of caregiving, shared caregiving responsibilities, and prior experience in caring for other family members.

#### 2.4.2. Family Assessment Device-General Function (FAD-GF)

The Chinese version of the Family Assessment Device-General Function (FAD-GF) was translated and adapted from the general function subscale of the Family Assessment Device, initially developed by Epstein, Baldwin, and Bishop in 1983 [28,29]. The Family Assessment Device comprises seven dimensions and a total of 60 questions [30]. This study employed the General Functional Subscale (FAD-GF), consisting of 12 questions, to evaluate the overall level of family function [28,29]. The internal consistency of the original Family Assessment Device, as assessed by Cronbach’s α, was reported as 0.92 [30]. For the Chinese version of the 12-item FAD-GF, the internal consistency, Cronbach’s α, was found to be 0.91 [28]. In Shek’s study [29], the internal consistency, Cronbach’s α, exceeded 0.80 and the test–retest reliability was 0.77 (*p* < 0.001). In this study, a Likert four-point scoring method was utilized, with 1 point indicating ‘strongly agree’ (best function) and 4 points indicating ‘strongly disagree’ (worst function). Some questions employed reverse scoring, specifically questions 1, 3, 5, 7, 9, and 11. For each question, responses of 1 or 2 suggested relatively healthy family function, while responses of 3 or 4 indicated potential impairment of family function. An average score exceeding 2 points suggested unhealthy overall family functioning. A higher score implied potential impairment of the overall operation of family function [31]. Cronbach’s α for the current study was 0.89.

#### 2.4.3. Caregiving Competence Scale-Chinese (CCS-C)

The Chinese version of the Caregiving Competence Scale (CCS-C) [32] was translated and adapted from the Caregiving Competence Scale (CCS) developed by Pearlin et al. [16]. This scale was utilized to assess the caregiving competence performance of family caregivers in the current study. The Cronbach’s α for the Chinese version of the CCS-C was found to be 0.81, and the kappa coefficient (κ) for the scale’s test–retest reliability ranged from 0.67 to 0.78. The scale comprised a total of four items, scored on a Likert 4-point scale from 1 (not at all) to 4 (very much). The total score ranged from a minimum of 4 points to a maximum of 16 points. A higher score indicated better caregiving competence. Cronbach’s α for the current study was 0.83.

#### 2.4.4. Ways of Coping Checklist-Revised (WCC-R)

The Ways of Coping Checklist-Revised (WCC-R), devised by Vitaliano, Russo, Carr, Mauiro, and Becker [33], was derived from coping scales initially developed by Lazarus and Folkmann [9]. The Chinese version of the WCC-R was translated from the Vitaliano version by Sheu [34]. This scale encompasses two coping strategies: problem-focused and emotion-focused. The content validity index (CVI) of the scale was reported to be 0.90, and the overall internal consistency, measured by Cronbach’s α, was found to be 0.80. Specifically, the Cronbach’s α for problem-focused coping was 0.79 and for emotion-focused coping, 0.67 [34]. In a study by Liang et al. [35] on brain tumor patients, Cronbach’s alpha for the overall scale was found to be 0.89, with 0.86 for problem-focused coping and 0.84 for emotion-focused coping. The scale comprised a total of 42 questions, utilizing a 5-point Likert scale from 0 to 4, from 0 (not used) to 4 (used a lot). A higher score on the scale indicated a more frequent use of the corresponding coping strategy. Cronbach’s α for the current study was 0.83.

### 2.5. Data Analysis

This study utilized the Statistical Package for the Social Sciences (SPSS) version 22.0 for Windows (SPSS, Chicago, IL, USA) for statistical analysis and data processing. Descriptive statistics, including mean, standard deviation, frequency, and percentage, were employed to examine the basic demographic characteristics of caregivers, general family functions, caregiver competencies, and coping strategies. The effects of different demographic attributes on caregivers’ coping strategies were evaluated using *t*-tests or one-way ANOVA. Pearson’s correlation was utilized to investigate the relationships among general family function, caregiver competencies, and coping strategies. Additionally, hierarchical multiple regression analysis was conducted to assess the predictive value of family general function and caregiver competencies on caregiver coping strategies.

## 3. Results

### 3.1. Demographics, Family Function, Caregiver Competence, and Coping Strategy

A total of 111 primary informal caregivers of brain tumor patients participated in this study. The patients had a mean age of 57.41 years (SD = 14.76, range 25–88), with 53.2% diagnosed with non-primary brain tumors, and 50.5% diagnosed more than 6 months ago. The majority of patients had cancer metastases (60.4%) and had undergone surgical treatment (78.4%), with 69.4% reporting no other chronic diseases. The mean Karnofsky performance status (KPS) score, reflecting their physical functional status, was 54.90 (SD = 18.68, range 10–100), suggesting that most patients (52.3%) had KPS scores higher than 60 (see Table 1).

The primary informal caregivers in this study had a mean age of 51.91 years (SD = 12.17, range 20–79), with 64.9% being female, 77.5% being married, and 60.4% having attained a college education or above. A considerable portion reported good (30.6%) or very good (30.6%) perceived health status, and 57.7% were employed. The majority of family caregivers were spouses (52.3%), and the primary type of care provided was shared caregiving (60.4%) (see Table 2).

The mean score for primary informal caregivers’ general family function was 1.89 (SD = 0.46, range: 1.00–3.58), with 64% scoring 2 points or less on average and 36% scoring more than 2 points. The mean score for primary caregivers’ caregiving competence was 2.71 (SD = 0.59, range: 1.00–4.00). Overall, the mean score for primary informal family caregivers’ coping was 2.55 (SD = 0.33, range: 1.76–3.36), with problem-focused coping averaging 2.92 (SD = 0.39, range: 1.81–3.67) and emotion-focused coping averaging 2.19 (SD = 0.49, range: 0.67–3.43).

### 3.2. Differences in Coping Strategies Based on the Demographics of Primary Informal Caregivers

The age of primary informal caregivers was related to significant differences in emotion-focused coping strategies (t = 2.01, *p* < 0.05) and total coping strategies (t = 2.35, *p* < 0.05). Additionally, the gender of primary informal caregivers (t = 2.21, *p* < 0.05) and their relationship with the patients (F = 3.14, *p* < 0.05) was related to significant differences in emotion-focused coping strategies. Male primary informal caregivers tended to have slightly higher scores in emotion-focused coping strategies compared to female counterparts. Among different categories of relationship with the patient, individuals classified as ‘others’ showed higher scores in utilizing emotion-focused coping compared to those identified as spouses or children. Moreover, the educational level of primary informal caregivers significantly impacted problem-focused coping scores (F = 8.56, *p* < 0.001) and total coping scores (F = 4.86, *p* < 0.05). Individuals with a diploma education or higher displayed notably better scores in problem-focused coping strategies than those with a high school education or below. Similarly, those with a diploma education or above also exhibited significantly higher total coping strategy scores compared to individuals with junior high school education or less. Furthermore, primary informal caregivers’ annual household income had a significant effect on problem-focused coping strategies (F = 7.69, *p* < 0.01). Those with higher annual household incomes demonstrated significantly higher scores in problem-focused coping strategies compared to those with lower incomes. Additionally, there was a significant difference in total coping strategies between caregivers with and without shared caregiving responsibilities (t = 2.10, *p* < 0.05). Primary informal caregivers without shared caregiving responsibilities exhibited significantly higher total coping strategy scores (see Table 2).

### 3.3. Relationship among Family Function, Caregiving Competence, and Coping Strategies of Primary Informal Caregivers

Pearson’s product–moment correlation analysis was employed to assess the relationships among primary informal caregivers’ family function, caregiving competence, and coping strategies. The results revealed a significant positive correlation between primary informal caregivers’ caregiving competence and problem-focused coping strategies (r = 0.242, *p* < 0.05). This suggests that higher levels of caregiving competence were associated with higher scores of problem-focused coping strategies. Conversely, a significant positive correlation was observed between primary caregivers’ family function and their emotion-focused coping strategies (r = 0.209, *p* < 0.05). This indicates that poorer family function was linked to higher scores in emotion-focused coping strategies (see Table 3).

### 3.4. Prediction of Coping Strategies among Primary Informal Caregivers Based on Demographics, Family Function, and Caregiving Competence

In this study, demographic characteristics such as age, gender, educational level, annual household income, kinship relationship, and shared caregiving responsibilities were found to significantly influence coping strategies among primary informal caregivers. Subsequently, hierarchical multiple regression analysis was conducted to assess the variance of these demographic characteristics, family function, and caregiving competence with primary informal caregivers’ problem-focused coping strategies, emotion-focused coping strategies, and total coping strategies. Collinearity assessment revealed a tolerance range of 0.65–0.95 and Variance Inflation Factor values ranging from 1.05 to 1.54, indicating no collinearity among the predictor variables.

In the hierarchical regression analysis, demographic characteristics were selected as predictor variables at the initial level, with family function chosen as the predictor at the subsequent level, followed by caregiving competence as the final predictor to forecast caregivers’ coping strategies. These predictors collectively accounted for a significant portion of the variance in primary caregivers’ problem-focused coping strategies, explaining 31% of the total variance (F = 5.737, *p* < 0.001). Specifically, caregiving competence contributed 2.80% to the variance in these coping strategies (F = 4.082, *p* < 0.05) (refer to Table 4).

In addition, these predictor variables collectively accounted for 18.20% of the total variance in the primary informal caregivers’ emotion-focused coping strategies (F = 2.841, *p* < 0.01). However, neither family function nor caregiving competence significantly predicted the primary informal caregivers’ emotion-focused coping strategies (see Table 5).

These predictor variables collectively explained 14.70% of the total variance in the primary informal caregivers’ total coping strategies (F = 2.194, *p* < 0.05). Nevertheless, neither family function nor caregiving competence significantly predicted the primary informal caregivers’ total coping strategies (see Table 6).

## 4. Discussion

Understanding coping strategies and their predictors in informal caregivers of family members with brain tumors can help professional caregivers identify ways to improve these strategies. This study aimed to explore the relationships among family function, caregiving competence, and coping strategies among primary informal caregivers. This study revealed a positive correlation between the family function of primary informal caregivers and their employment of emotion-focused coping. Additionally, the caregiving competence of primary informal caregivers exhibited a significant association with their use of problem-focused coping. Specifically, caregiving competence emerged as a significant predictor of problem-focused coping. However, neither family function nor caregiving competence demonstrated a significant correlation with primary caregivers’ total coping strategies.

In terms of coping strategies, this study revealed that primary informal caregivers exhibited higher scores for problem-focused coping compared to emotion-focused coping. This observation aligns with the results reported by Chen et al. [36] and Liang et al. [35] in their studies involving Taiwanese cancer patients, as well as with the findings of Coppetti et al. [10] in their research involving primary caregivers of cancer patients. These studies consistently reported higher scores for problem-focused coping than for emotion-focused coping. Similarly, Baumstarck et al. [37] investigated primary family caregivers of patients with highly malignant glioma and also observed higher scores for problem-focused coping. According to Lazarus and Folkman’s stress coping theory [9], individuals tend to employ problem-focused coping when they perceive a stressful situation as within their control or amenable to change. Conversely, when individuals perceive a stressful situation as beyond their control or unchangeable, they are more likely to utilize emotion-focused coping. However, both problem-focused and emotion-focused coping serve potential functions, regardless of which approach an individual chooses to adopt [9].

The present study’s higher scores in problem-focused coping compared to emotion-focused coping may be attributed to caregivers’ perception of possessing the capabilities necessary to manage situations related to the home care of brain tumor patients. Despite 47.7% of patients in this study having a KPS score indicating a need for considerable assistance and frequent medical care or even severe physical dysfunction requiring specialized treatment, hospitalization, or assistance, most patients had KPS scores exceeding 60 points, indicating a level of functionality that surpasses the need for occasional assistance but still allows for the management of most personal needs. This suggests that patients’ physical functional status, as indicated by the KPS, indirectly and partially explains why family caregivers, despite facing caregiving pressures, still felt equipped to manage most situations.

The findings of this study revealed a significant positive correlation between the family function of primary informal caregivers and their use of emotion-focused coping. However, no correlation was observed between family function and problem-focused coping. Shao, Zhong, Wu, Yan, and Zhang [15] found that, in addition to family function being significantly positively correlated with positive coping, positive coping also serves as a mediating variable between family function and related outcomes. Research by Kim and Ahn [38] also found that family functioning was significantly positively related to both problem-solving communication and coping. While some studies focusing on younger populations demonstrated that family function can significantly predict individual coping strategies [15,26], research by Hanksa, Rapportc, and Vangel [39] examining various relationships of family caregivers of patients with brain injuries found no association between family function and caregivers’ coping strategies. This discrepancy may arise from differences in study populations or the severity and type of the diseases being cared for by the caregivers. For example, Kim and Ahn [38] focused their research on the relationships between spouses and families affected by gynecologic cancer. However, the subjects of the current study included not only spouses but also other relationships, such as those with children. Therefore, the role of family function in shaping individual coping strategies may vary.

The assessment of family function in this study encompassed various aspects, including interaction, support, and decision-making among family members within the family unit. A lower score on the family function scale indicated healthier family function. Most family caregivers (64%) reported positive family function, with a mean score below two points. However, the results of this study revealed that caregivers experiencing poorer family function tended to rely more on emotion-focused coping. It is important to note that coping strategies are not inherently good or bad [9]. In circumstances where family function is compromised, emotion-focused coping strategies may assume greater importance in helping family members navigate the stress they perceive.

The findings of this study indicate a significant positive correlation between the caregiving competence of primary informal caregivers and their utilization of problem-focused coping, with caregiving competence significantly predicting problem-focused coping. This observation aligns with the findings of previous research on primary informal caregivers of cancer patients [10,14]. In those studies, caregiving competence, encompassing the three facets of knowledge, courage, and patience, revealed a notable and significant correlation with problem-focused coping, particularly with the knowledge aspect. However, it is noteworthy that, in this study, caregiving competence was defined specifically as the assessment of family caregivers’ ability to perform caregiving tasks. Consequently, higher caregiving competence may indicate that caregivers possess relevant caregiving knowledge or that the caregiving tasks are not excessively challenging or burdensome. Hence, the perceived stress experienced by caregivers, or their assessment of this pressure, may not necessarily be harmful or dangerous, thereby influencing their choice of coping strategies. This may involve seeking further pertinent information or adjusting the relevant circumstances [40], demonstrating a preference for problem-focused coping.

The primary predictor of problem-focused coping among family caregivers in this study was their caregiving competence. Consequently, healthcare professionals should prioritize efforts to enhance the caregiving competence of family caregivers. A systematic review and meta-analysis by Bayly et al. [41] demonstrated the efficacy of various interventions in strengthening the caregiving competence of family members, including psychoeducational interventions and early-stage interventions. Notably, individual interventions were found to be more effective than group-based interventions. For instance, Cheng, Chair, and Chau [42] conducted a strength-oriented psychoeducational program that not only improved the caregiving competence of family members but also enhanced their problem-solving coping abilities. This program comprised components including affective experiencing, aimed at guiding family caregivers to leverage positive emotions in the face of caregiving tasks, behavioral regulation to guide family caregivers to modify caregiving skills and habits to promote their own well-being, and cognitive mastery to guide family caregivers to reshape their perception of the problem and develop problem-solving skills and abilities.

Healthcare professionals should also consider the impact of demographic factors such as gender, age, education level, and financial income on the coping strategies of family caregivers. Specifically, males, younger individuals, those with lower levels of education, and those with lower incomes are more inclined to rely on emotion-focused coping. It is possible that caregivers with these characteristics often encounter caregiving situations where they feel powerless to effect change. Therefore, healthcare professionals should prioritize support and guidance for these groups, aiding them in acquiring pertinent caregiving knowledge and skills.

This study adopted a cross-sectional correlational design utilizing convenience sampling. The sample was drawn solely from a neurosurgery ward within a medical center in northern Taiwan, potentially limiting the generalizability of the findings. Moreover, given the nature of cross-sectional studies, the results are confined to a specific time point, and, while regression analysis was employed to investigate predictors, causal inferences cannot be made. Conducting a longitudinal study in the future would offer a more comprehensive understanding of changes in patients’ disease conditions, as well as fluctuations in family function, caregiving competence, and coping strategies among primary informal caregivers.

## 5. Conclusions

The findings of this study suggest that primary informal caregivers of brain tumor patients tend to employ problem-focused coping strategies, and improving the caregiving competence of these caregivers could promote the adoption of problem-focused coping strategies. In addition to strength-oriented psychoeducational programs that can increase the caregiving competence of family caregivers, group discussions about their experiences and challenges in communicating with patients, combined with role-playing strategies to enhance communication, can be effective for problem-solving [43]. Additionally, planning and strategic thinking in developing solutions for specific problems are crucial components of effective problem-solving skills [44]. It is essential for healthcare professionals to strengthen the caregiving competence of family caregivers, as this can support their adoption of problem-focused coping strategies. In particular, healthcare professionals should pay more attention to groups that predominantly use emotion-focused coping strategies, as they may be less able to control or change the care situation. Future research efforts could focus on designing interventions to enhance the caregiving competence of family members caring for brain tumor patients. Such interventions are likely to empower family caregivers to develop effective coping strategies for managing the challenges associated with caregiving.

## Figures and Tables

**Table 1:** Demographic and medical characteristics of patients (*N* = 111).

Variable	*n*	%
Age		
<60 years	59	53.2
≧60 years	52	46.8
Gender		
Male	58	52.3
Female	53	47.7
Brain tumor diagnosis		
Primary	52	46.8
Non-primary	59	53.2
Time since brain tumor diagnosis		
>6 months	56	50.5
≦6 months	55	49.5
Brain tumor metastasis		
Yes	67	60.4
No	44	39.6
Surgical treatment		
Yes	87	78.4
No	24	21.6
Other treatment		
Yes	61	55.0
No	50	45.0
Other chronic disease		
Yes	34	30.6
No	77	69.4
Physical functional status (KPS)		
≦60	53	47.7
>60	58	52.3

KPS: Karnofsky performance status.

**Table 2:** Demographic characteristics of caregivers by coping strategies (*N* = 111).

Variable			Problem-Focused	Emotion-Focused	Total
	*n*	%	t/F, M(SD)	t/F, M(SD)	t/F, M(SD)
Age			1.36	2.01 *	2.35 *
<60 years	79	71.2	2.95(0.37)	2.24(0.48)	2.59(0.30)
≧60 years	32	28.8	2.83(0.41)	2.04(0.47)	2.44(0.35)
Gender			−1.64	2.21 *	0.66
Male	39	35.1	2.83(0.37)	2.32(0.44)	2.53(0.34)
Female	72	64.9	2.96(0.38)	2.11(0.49)	2.53(0.31)
Education Level			8.56 *** ③ > ①, ③ > ②	1.88	4.86 * ③ > ①
①Junior high school and under	20	18	2.69(0.41)	2.02(0.47)	2.36(0.35)
②Senior high school	24	21.6	2.78(0.36)	2.31(0.41)	2.54(0.34)
③Diploma and higher	67	60.4	3.03(0.35)	2.19(0.51)	2.61(0.29)
Marital Status			0.89	−0.54	0.12
Married	86	77.5	2.93(0.39)	2.17(0.49)	2.55(0.34)
Others	25	22.5	2.85(0.34)	2.23(0.48)	2.54(0.26)
Religion affiliation			1.15	0.98	1.05
Buddhism	55	49.5	2.93(0.39)	2.12(0.44)	2.53(0.31)
Taoism	27	24.3	2.97(0.31)	2.28(0.52)	2.63(0.34)
Other	29	26.1	2.82(0.43)	2.21(0.53)	2.52(0.35)
Employment status			−0.57	−1.41	−1.41
Unemployed	47	42.3	2.89(0.39)	2.11(0.52)	2.50(0.35)
Employed	64	57.7	2.93(0.38)	2.24(0.45)	2.58(0.29)
Annual household income			7.69 ** ③ > ①, ② > ①	0.90	1.07
500,000	32	28.8	2.72(0.38)	2.28(0.54)	2.50(0.37)
510,000–1 million	41	36.9	2.93(0.35)	2.13(0.44)	2.53(0.31)
More than 1.01 million	38	34.2	3.06(0.36)	2.15(0.49)	2.61(0.29)
Health status			1.63	1.13	1.15
Extremely good	14	12.6	2.98(0.37)	2.08(0.56)	2.53(0.31)
Very good	34	30.6	3.02(0.29)	2.22(0.45)	2.62(0.26)
Good	34	30.6	2.88(0.40)	2.16(0.49)	2.52(0.34)
Average	24	21.6	2.80(0.47)	2.30(0.52)	2.54(0.39)
Poor	5	4.5	2.79(0.26)	1.84(0.29)	2.31(0.21)
Kinship relationship			0.21	3.14 * ③ > ①, ③ > ②	1.08
Spouse	58	52.3	2.92(0.37)	2.12(0.44)	2.53(0.33)
Child	27	24.3	2.93(0.44)	2.11(0.51)	2.52(0.31)
Other	26	23.4	2.87(0.36)	2.39(0.52)	2.63(0.35)
Type of caregiving			−0.54	−0.74	−0.88
Shared caregiving	67	60.4	2.90(0.42)	2.15(0.51)	2.53(0.33)
Independent caregiving	44	39.6	2.94(0.32)	2.22(0.45)	2.58(0.31)
Caregiving duration			0.20	1.01	0.88
Full time	62	55.9	2.92(0.39)	2.22(0.44)	2.57(0.32)
Rotation	49	44.1	2.91(0.38)	2.13(054)	2.52(0.33)
Shared caregiving responsibilities			1.27	1.76	2.10 *
No	43	38.7	2.98(0.35)	2.29(0.45)	2.63(0.31)
Yes	68	61.3	2.88(0.41)	2.12(0.50)	2.50(0.32)
Caregiving experience			1.10	−0.49	0.28
None	68	61.3	2.95(0.37)	2.16(0.54)	2.55(0.34)
Yes	43	38.7	2.86(0.40)	2.21(0.38)	2.54(0.30)

Note: *p* < 0.05 *, *p* < 0.01 **, *p* < 0.001 ***.

**Table 3:** Relationship among family function, caregiving competence, and coping strategies of family caregivers (*N* = 111).

Variable	Problem-Focused	Emotion-Focused	Total Coping Strategy
Family Function	−0.165	0.209 *	0.059
Caregiving Competence	0.242 *	−0.149	0.031

Note: * *p* < 0.05.

**Table 4:** Prediction of problem-focused coping strategies among primary informal caregivers based on demographics, family function, and caregiving competence (*N* = 111).

Level	Variable	Estimated B Value	Standard Error	β Coefficient	R^2^	Change in R^2^	Changes in F Value
Level 1	Age	−0.030	0.080	−0.035	0.267	0.267	6.327 ***
	Gender	0.211	0.068	0.262 **			
	Education level	0.139	0.051	0.280 **			
	Annual household income	0.120	0.047	0.247 *			
	Kinship relationship	−0.027	0.041	−0.057			
	Sharing of caregiving responsibilities	−0.127	0.067	−0.161			
Level 2	Family function	−0.056	0.079	−0.065	0.283	0.015	2.199
Level 3	Caregiving Competence	0.119	0.059	0.182 *	0.310	0.028	4.082 *
Overall Model R^2^ = 0.310 (*F*(8, 102) = 5.737, *p* = 0.000)							

Note: * indicates *p* < 0.05; ** indicates *p* < 0.01; *** indicates *p* < 0.001.

**Table 5:** Prediction of emotion-focused coping strategies among primary informal caregivers based on demographics, family function, and caregiving competence (*N* = 111).

Level	Variable	Estimated B Value	Standard Error	β Coefficient	R^2^	Change in R^2^	Change in F Value
Level 1	Age	−0.169	0.110	−0.157	0.158	0.158	3.261 **
	Gender	−0.213	0.094	−0.208 *			
	Education Level	0.016	0.070	0.025			
	Annual Household Income	−0.078	0.065	−0.127			
	Kinship Relationship	0.095	0.057	0.159			
	Shared Caregiving Responsibilities	−0.180	0.092	−0.179			
Level 2	Family Function	0.093	0.109	0.086	0.172	0.014	1.747
Level 3	Caregiving Competence	−0.090	0.081	−0.109	0.182	0.010	1.229
Overall Model R^2^ = 0.182 (*F*(8, 102) = 2.841, *p* = 0.007)							

Note: * indicates *p* < 0.05; ** indicates *p* < 0.01;

**Table 6:** Prediction of total coping strategies among primary informal caregivers based on demographics, family function, and caregiving competence (*N* = 111).

Level	Variable	Estimated B Value	Standard Error	β Coefficient	R^2^	Change in R^2^	Change in F Value
Level 1	Age	−0.099	0.075	−0.139	0.146	0.146	2.963 *
	Gender	−0.001	0.064	−0.001			
	Education Level	0.077	0.047	0.185			
	Annual Household Income	0.021	0.044	0.052			
	Kinship Relationships	0.034	0.038	0.086			
	Shared Caregiving Responsibilities	−0.154	0.062	−0.231 *			
Level 2	Family Function	0.019	0.074	0.026	0.146	0.000	0.030
Level 3	Caregiving Competence	0.014	0.055	0.026	0.147	0.001	0.069
Overall Model R^2^ = 0.147 (*F*(8, 102) = 2.194, *p* = 0.034)							

Note: * indicates *p* < 0.05.

## Data Availability

Data presented in this study are available from the first author upon reasonable request.

## References

[1] Centers for Disease Control and Prevention (2020). United States Cancer Statistics: Data Visualizations. https://gis.cdc.gov/Cancer/USCS/DataViz.html.

[2] American Cancer Society (2019). Cancer Fact & Figures. https://www.cancer.org/content/dam/cancer-org/research/cancer-facts-and-statistics/annual-cancer-facts-and-figures/2019/cancer-facts-and-figures-2019.pdf.

[3] Health Promotion Administration CANCER REGISTRY ANNUAL REPORT (p. 94), 2021 TAIWAN. https://www.hpa.gov.tw/File/Attach/17639/File_23506.pdf.

[4] Armstrong T.S., Vera-Bolanos E., Acquaye A.A., Gilbert M.R., Ladha H., Mendoza T. (2016). The symptom burden of primary brain tumors: Evidence for a core set of tumor- and treatment-related symptoms. Neuro-Oncology.

[5] IJzerman-Korevaar M., Snijders T.J., de Graeff A., Teunissen S., de Vos F.Y.F. (2018). Prevalence of symptoms in glioma patients throughout the disease trajectory: A systematic review. J. Neurooncol..

[6] van Grinsven E.E., Smits A.R., van Kessel E., Raemaekers M.A.H., de Haan E.H.F., Huenges Wajer I.M.C., Ruijters V.J., Philippens M.E.P., Verhoeff J.J.C., Ramsey N.F. (2023). The impact of etiology in lesion-symptom mapping—A direct comparison between tumor and stroke. Neuroimage Clin..

[7] Lemaitre A.L., Herbet G., Duffau H., Lafargue G. (2021). Personality and behavioral changes after brain tumor resection: A lesion mapping study. Acta Neurochir..

[8] Kershaw T., Northouse L., Kritpracha C., Schafenacker A., Mood D. (2004). Coping strategies and quality of life in women with advanced breast cancer and their family caregivers. Psychol. Health.

[9] Lazarus R.S., Folkman S. (1984). Stress, Appraisal, and Coping.

[10] Coppetti L.D.C., Girardon-Perlini N.M.O., Andolhe R., Silva L.M.C.D., Dapper S.N., Noro E. (2019). Caring ability, burden, stress and coping of family caregivers of people in cancer treatment. Rev. Bras. Enferm..

[11] Folkman S., Greer S. (2000). Promoting psychological well-being in the face of serious illness: When theory, research and practice inform each other. Psycho-Oncology.

[12] Bowman K.F., Rose J.H., Radziewicz R.M., O’Toole E.E., Berila R.A. (2009). Family caregiver engagement in a coping and communication support intervention tailored to advanced cancer patients and families. Cancer Nurs..

[13] Liang S.Y., Chang T.T., Wu W.W., Wang T.J. (2019). Caring for patients with oral cancer in Taiwan: The challenges faced by family caregivers. Eur. J. Cancer Care.

[14] Siqueira F.D., Girardon-Perlini N.M.O., Andolhe R., Zanini R.R., Santos E.B.D., Dapper S.N. (2021). Caring ability of urban and rural family caregivers: Association with overburden, stress and coping. Rev. Esc. Enferm. USP.

[15] Shao L., Zhong J.D., Wu H.P., Yan M.H., Zhang J.E. (2022). The mediating role of coping in the relationship between family function and resilience in adolescents and young adults who have a parent with lung cancer. Support. Care Cancer.

[16] Pearlin L.I., Mullan J.T., Semple S.J., Skaff M.M. (1990). Caregiving and the stress process: An overview of concepts and their measures. Gerontologist.

[17] Olson D.H. (2000). Circumplex model of marital and family systems. J. Fam. Ther..

[18] Epstein N.B., Ryan C.E., Bishop D.S., Miller I.W., Keitner G.I., Walsh F. (2003). The McMaster model: A view of healthy family functioning. Normal Family Processes: Growing Diversity and Complexity.

[19] Cuhadar D., Savas H.A., Unal A., Gokpınar F. (2015). Family Functionality and Coping Attitudes of Patients with Bipolar Disorder. J. Relig. Health.

[20] Hamid P.N., Yue X.D., Leung C.M. (2003). Adolescent coping in different Chinese family environments. Adolescence.

[21] Shao R., He P., Ling B., Tan L., Xu L., Hou Y., Kong L., Yan Y. (2020). Prevalence of depression and anxiety and correlations between depression, anxiety, family functioning, social support and coping styles among Chinese medical students. BMC Psychol..

[22] Ren Y., Lin M.P., Liu Y.H., Zhang X., Wu J.Y.W., Hu W.H., Xu S., You J. (2018). The mediating role of coping strategy in the association between family functioning and nonsuicidal self-injury among Taiwanese adolescents. J. Clin. Psychol..

[23] Dusek J.B., Danko M. (1994). Adolescent coping styles and perceptions of parental child rearing. J. Adolesc. Res..

[24] Huss M., Lehmkuhl U. (1996). Coping in the family context: Active and avoidance strategies in adolescents from divorced families. Prax. Kinderpsychol. Kinderpsychiatr..

[25] Kliewer W., Lewis H. (1995). Family influences on coping processes in children and adolescents with sickle cell disease. J. Pediatr. Psychol..

[26] Chen Y.A. (2014). The Relationship between Family Functioning and Depression among High Concern Student: The Mediating Role of Coping Strategy. Master’s Thesis.

[27] Cuschieri S. (2019). The STROBE guidelines. Saudi J. Anaesth..

[28] Li R., Xu F., Ji L. (2013). Revision of Family Assessment Device(FAD). China J. Health Psychol..

[29] Shek D.T.L. (2001). The General Functioning Scale of the Family Assessment Device: Does it work with Chinese adolescents?. J. Clin. Psychol..

[30] Epstein N.B., Baldwin L.M., Bishop D.S. (1983). The McMaster Family Assessment Device. J. Marital. Fam. Ther..

[31] Miller I.W., Ryan C.E., Keitner G.I., Bishop D.S., Epstein N.B. (2000). The McMaster approach to families: Theory, assessment, treatment and research. J. Fam. Ther..

[32] Cheng H.Y., Chair S.Y., Chau J.P.C. (2017). Psychometric evaluation of the Caregiving Competence Scale among Chinese family caregivers. Rehabil. Nurs. J..

[33] Vitaliano P.P., Russo J., Carr J.E., Maiuro R.D., Becker J. (1985). The ways of coping checklist: Revision and psychometric properties. Multivar. Behav. Res..

[34] Sheu S.L. (2001). Uncertainty and anxiety in patients with initial attack of myocardial infarction: The effect of coping methods. J. Nurs. Res..

[35] Liang S.Y., Liu H.C., Lu Y.Y., Wu S.F., Chien C.H., Tsay S.L. (2020). The Influence of Resilience on the Coping Strategies in Patients with Primary Brain Tumors. Asian Nurs. Res..

[36] Chen S.C., Wu S.F.V., Wang T.J., Rosenberg J., Lu Y.Y., Liang S.Y. (2022). Factors Influencing the Coping Strategies of Liver Cancer Patients Undergoing Transarterial Chemoembolization. Int. J. Nurs. Pract..

[37] Baumstarck K., Leroy T., Hamidou Z., Tabouret E., Farina P., Barrie M., Campello C., Petrirena G., Chinot O., Auquier P. (2016). Coping with a newly diagnosed high-grade glioma: Patient-caregiver dyad effects on quality of life. J. Neuro-Oncol..

[38] Kim M., Ahn S. (2022). Do spouse burden of care, family resilience, and coping affect family function in gynecologic cancer in Korea?: A cross-sectional study. Korean J. Women Health Nurs..

[39] Hanksa R.A., Rapportc L.J., Vangel S. (2007). Caregiving appraisal after traumatic brain injury: The effects of functional status, coping style, social support and family functioning. NeuroRehabilitation.

[40] Folkman S., Lazarus R.S., Gruen R.J., DeLongis A. (1986). Appraisal, Coping, Health Status, and Psychological Symptoms. J. Pers. Soc. Psychol..

[41] Bayly M., Morgan D., Elliot V., Kosteniuk J., Chow A.F., Peacock S., O’Connell M.E. (2021). Does early-stage intervention improve caregiver well-being or their ability to provide care to persons with mild dementia or mild cognitive impairment? A systematic review and meta-analysis. Psychol. Aging.

[42] Cheng H.Y., Chair S.Y., Chau J.P.C. (2018). Effectiveness of a strength-oriented psychoeducation on caregiving competence, problem-solving abilities, psychosocial outcomes and physical health among family caregiver of stroke survivors: A randomised controlled trial. Int. J. Nurs. Stud..

[43] Caga J., Zoing M.C., Foxe D., Ramsey E., D’Mello M., Mioshi E., Ahmed R.M., Kiernan M.C., Piguet O. (2021). Problem-focused coping underlying lower caregiver burden in ALS-FTD: Implications for caregiver intervention. Amyotroph. Lateral Scler. Front. Degener.

[44] Ghane G., Farahani M.A., Seyedfatemi N., Haghani H. (2016). Effectiveness of problem-focused coping strategies on the burden on caregivers of hemodialysis patients. Nurs. Midwifery Stud..

